# Enhancing the Fatigue Strength of the Weld Line in Advanced Polymer Injection Molding: Gas-Assisted Mold Temperature Control for Thermoplastic Polyurethane (TPU) Composites

**DOI:** 10.3390/polym15112440

**Published:** 2023-05-25

**Authors:** Pham Son Minh, Tran Minh The Uyen, Thanh Trung Do, Van-Thuc Nguyen, Van Thanh Tien Nguyen

**Affiliations:** 1 Faculty of Mechanical Engineering, Ho Chi Minh City University of Technology and Education, Ho Chi Minh City 71307, Vietnam; 2 Faculty of Mechanical Engineering, Industrial University of Ho Chi Minh City, Nguyen Van Bao Street, Ward 4, Go Vap District, Ho Chi Minh City 70000, Vietnam

**Keywords:** fatigue cycle, deformation, amplitude, frequency, injection molding

## Abstract

This study presents an innovative approach to enhancing weld line strength in advanced polymer injection molding through applying gas-assisted mold temperature control, significantly increasing mold temperature beyond typical values observed in conventional processes. We investigate the effects of various heating times and frequencies on the fatigue strength of Polypropylene (PP) samples and the tensile strength of Acrylonitrile Butadiene Styrene (ABS) composite samples at different Thermoplastic Polyurethane (TPU) percentages and heating times. Using gas-assisted mold heating, mold temperatures exceeding 210 °C are achieved, which represents a significant advancement compared to the standard mold temperatures of less than 100 °C. As a result, the fatigue strength of the PP sample with mold heating at 15 s shows a remarkable increase of up to 5.4 times at 5 Hz compared to the sample without mold temperature control. Moreover, ABS/TPU blends with 15 wt.% TPU exhibit the highest ultimate tensile strength (UTS) value of 36.8 MPa, while blends with 30 wt.% TPU have the lowest UTS value of 21.3 MPa. This advancement demonstrates the potential for improved welding line bonding and fatigue strength in manufacturing. Our findings reveal that increasing the mold temperature before injection results in higher fatigue strength in the weld line, with the TPU percentage having a more significant influence on the mechanical properties of ABS/TPU blends than heating time. The results of this study contribute to a deeper understanding of advanced polymer injection molding and offer valuable insights for process optimization.

## 1. Introduction

Injection molding is a prominent process in the plastics industry [1,2,3]. The hot melt plastic is pressed into the mold cavity during the injection. As the plastic transfers heat to the mold, its temperature slowly drops, and the material solidifies. However, rapid cooling at this stage may result in a reduction in product flow length. This phenomenon, in particular, can cause a short-fill problem in thin-walled products [4,5,6,7]. Furthermore, rapid cooling also weakens the product’s weld line.

To reduce the cooling rate of the hot melt plastic, mold temperature must be controlled via a heat-assisted system, such as gas-assisted mold temperature control, high-frequency-induced heating, and steam heating. Mold temperature can be controlled through heating either the inside or outside of the mold [8,9,10]. Managing the mold surface is easier and more efficient than maintaining the mold core. For example, Chen et al. [11] used gas-assisted mold temperature control to raise the temperature of the mold. They demonstrated that a heating rate of 28 °Cs^−1^ can raise the mold temperature to 229 °C. Giang et al. [12] applied external gas-assisted mold temperature control to the cavity surface. According to this study, the mold’s temperature could rise as high as 332.3 °C. A heating rate of 7.6 °Cs^−1^ could be achieved using high-frequency-induced heating in conjunction with a water cooling system [13].

**Figure 1:**
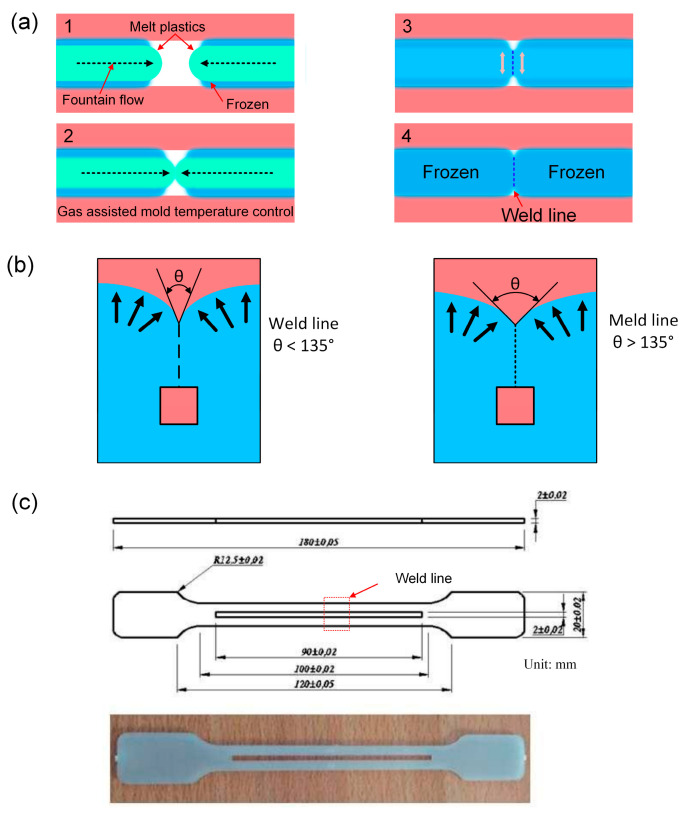
Weld line formation and internal gas-assisted mold temperature control: (**a**) weld line in injection molding, (**b**) weld line and meld line, and (**c**) injection sample size and shape.

Interestingly, Jeng et al. [14] used steam heating to control the mold temperature to improve the surface quality of TV housing molds. Uyen et al. [15] discovered that the first 20 s of gas-assisted mold temperature control are more efficient than the later period. This gas-assisted mold temperature control uses hot gas supported with 12-kW power, with outside dimensions of 240 × 100 × 60 mm. Ambient air was allowed to flow into the air drier at 0.7 MPa. The heated air flowed through the gas gate, with a hole diameter of 10 mm. The high-power hot gas generator system could provide a flow of hot air reaching 400 °C. With this hot gas system, the mold can be heated at 19.6 °Cs^−1^. According to Roth et al. [16], the plate aspect ratio could be increased to 125% using a high-pressure press and a dynamic mold temperature control system. Controlling the mold’s temperature could also minimize the weld line’s effects. Weld lines in injection molding are frequently unavoidable. Figure 1a illustrates how the weld line is produced when two melt streams merge during the final filling process. Weld line existence reduces the material strength; however, its demerit characteristics can be eliminated. Many prior studies tried to enhance the weld line strength [17,18,19,20,21]—the weld line forms when two molten streams meet at an angle smaller than 135°. Besides the weld line, a meld line could also be formed during the solidification process when two molten streams meet each other at an angle larger than 135°, as shown in Figure 1b. Fathi et al. [22] examined the flow behavior to observe the weld line and meld line formation. This study indicated that the meld line area has better mechanical properties than the weld line area. Ozcelik et al. [23] pointed out that weld line and meld line reduces the ductility of the polymer significantly. Moayyedian et al. [24] designed a novel edge gate to avoid the formation of weld lines, meld lines, and sink marks.

Injection molding could be applied with many polymer materials, such as polypropylene (PP), polyethylene (PE), polystyrene (PS), acrylonitrile butadiene styrene (ABS), polyethylene terephthalate (PET), thermoplastic polyurethane (TPU), and polyvinyl chloride (PVC) [25,26,27,28,29]. Among them, ABS and PP are commonly used as they have excellent characteristics of low cost, lightweight, good strength, and toughness [30,31]. Plastic products often suffer a lower rate of loading than alloy products. However, cyclic loads can cause severe damage due to fatigue failure in materials [32]. This phenomenon reduced the durability of plastic products. As a result, increasing fatigue strength, particularly at the weld line, is critical to improving product quality. Glass fiber and additives can be used to improve fatigue strength [33,34,35,36,37]. These methods, however, usually result in higher costs and lower toughness. Normally, plastic products are generated in pure condition with minimum additives.

On the other hand, controlling mold temperature could lower the rate of frozen parts during injection molding. The weld line tensile strength of PP is investigated by Wu et al. [38], and the fatigue strength of the weld line could be increased as a result. This strategy, though, is rarely discussed.

TPU has a high adhesive capacity; thus, PP and ABS can be combined to strengthen the bonding of the blends, especially where the weld line is concerned. The PP/TPU blend characteristics are examined by Lou et al. [39], Nguyen et al. [40], and Kannan et al. [41]. They indicated that when mixing well and adding nanoclay, the mechanical properties of the blend are improved. They combined 10–30 wt.% TPU and ABS to improve layer adhesion due to hydrogen bonding between molecules, according to De León et al. [42]. As a result, the strength between layers is increased, as is the strength between layers and the platform. However, the weld line tensile strength of ABS/TPU blends is not thoroughly investigated, especially when injected at a high mold temperature. Moreover, the assistance of pre-heating could also enhance the weld line strength of PP polymer, and these issues are not investigated in detail.

This report uses gas-assisted mold temperature control to support the injection process of PP and ABS/TPU samples. The fatigue strength of polypropylene (PP) samples is investigated with regard to various heating times and frequencies. Moreover, different TPU percentages and heating times are used to evaluate the tensile strength of ABS/TPU samples. This study seeks to identify ways to optimize the injection molding process and extend the lifespan of plastic goods. The findings of this research may be valuable in various industries, ranging from automotive production to consumer goods, where injection molding is a standard manufacturing process.

## 2. Experimental Methods

### 2.1. Materials

In this study, the ABS 750 SW polymer was manufactured by Kumho Petrochemical, Seoul, Korea, with a MFR/MVR of 38 g/10 min and a Vicat softening point of 95 °C. The TPU polymer (TPU^®^ TU90AE) was manufactured by Dongguan Rayan Polymer, Dongguan, China, with a MFR/MVR of 79 g/10 min and a Vicat softening point of 85.5 °C. The PP polymer (Advanced-PP 1100 N) was manufactured by Advanced Petrochemical Company, Al Jubail, Saudi Arabia, with the MFR/MVR and the Vicat softening point being 12 g/10 min and 154 °C, respectively. Five heating time levels were used to explore the effects of heating time on PP samples: 0, 3, 6, 9, and 12 s. Moreover, to investigate the impact of TPU percentage and heating time on the characteristics of ABS/TPU composite, TPU percentages were set at five levels: 10, 15, 20, 25, and 30 wt.%. At the same time, the heating time was set at five levels: 0 s, 3 s, 6 s, 9 s, and 12 s. The injected sample shape follows the ASTM D638 standards, as shown in Figure 1c.

### 2.2. Procedures of Composite

**Figure 2:**
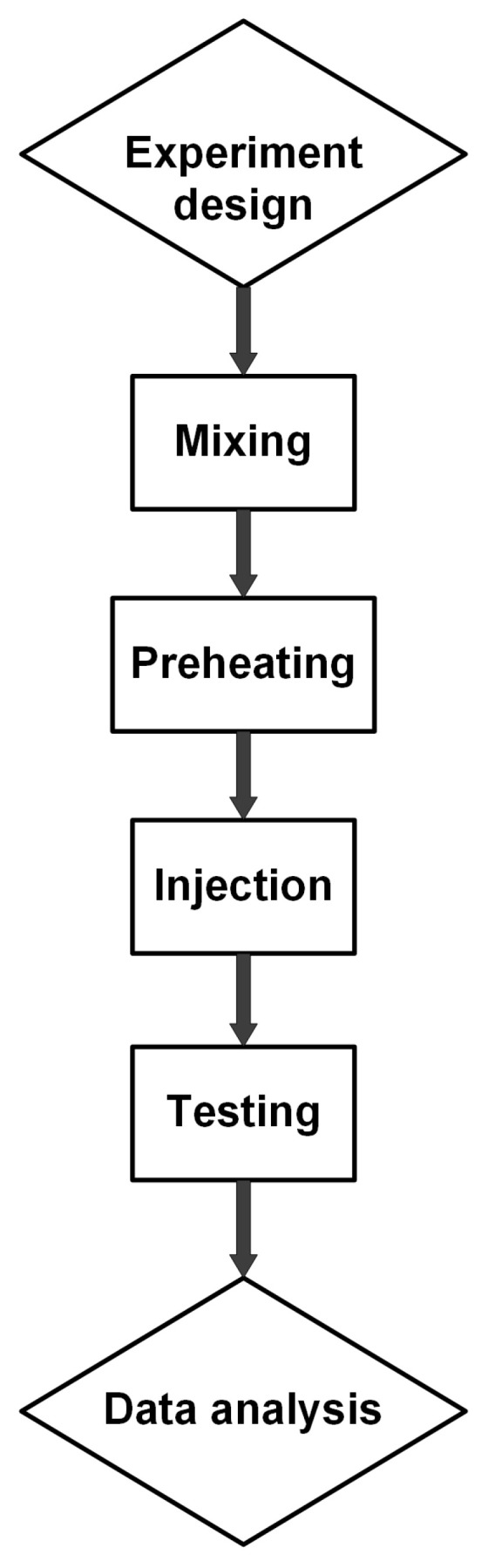
General schematic diagram of experiment.

**Figure 3:**
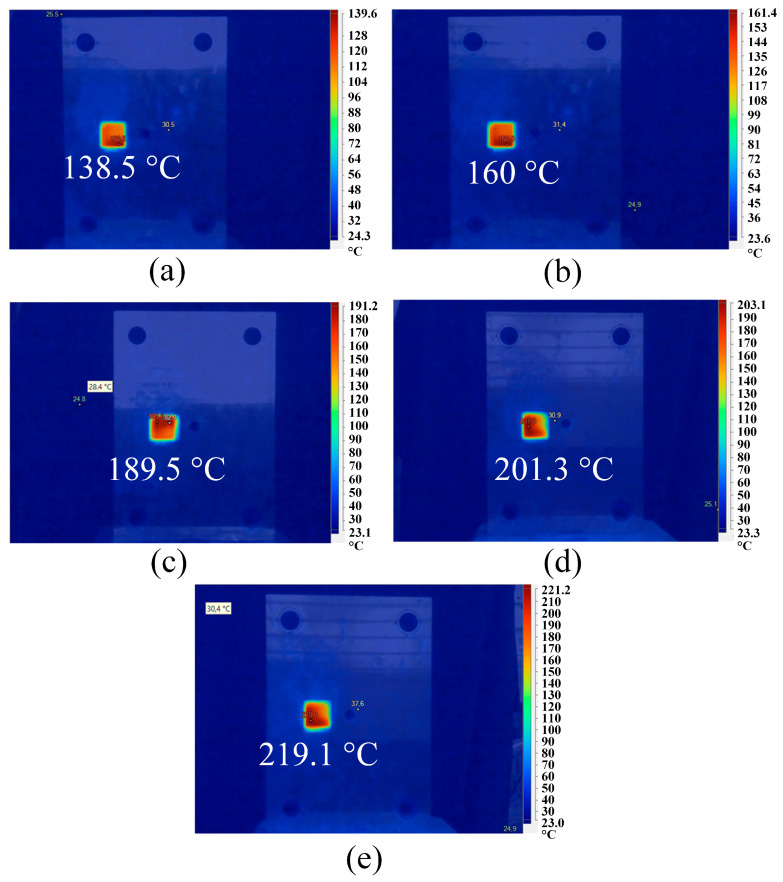
Average mold temperature at weld line area at different heat times: (**a**) 3 s, (**b**) 6 s, (**c**) 9 s, (**d**) 12 s, and (**e**) 15 s.

Figure 2 shows the general schematic diagram of the experiment. The injection molding machine was Haitian-MA 1200III. Before injection molding, the mold was pre-heated with a Makita HG6530V heat gun. The heating gun was set at 600 °C hot air, along with an airflow of 550 l/min and a distance of 10 mm. The distance was fixed using a fixture clamp. The mold temperature was checked using a Fluke TiS20 infrared camera (Fluke Corporation, Everett, Washington, DC, USA). Figure 3 shows the mold temperature at different heat times. The mold temperature values were 138.5, 160, 189.5, 201.3 and 219.1 °C, corresponding to 3, 6, 9, 12, and 15 s. For every 3 s, the mold temperature can increase by about 20 °C—PP polymer pellets with 5 wt.% CaCO_3_ for improving the mechanical properties were dried before injection. After that, they were injected into a pre-heated mold with different pre-heat temperatures in the molding condition presented in Table 1. Table 1 shows the initial temperature of 70 °C because this was the mold temperature used during the normal injection molding process. The heat from the injection molding process with multi-time injections led to a mold temperature that was higher than the ambient temperature. Therefore, the initial temperature of the mold was selected due to the effects of the heat after some initial injections. The first 3 s heated the mold to about 68.5 °C, which was much quicker than the heating from 3 s to 6 s. The reason for this rapid heating was the characteristics of heat transformation from hot air to steel mold. The heat transfer efficiency reduced when the mold temperature increased. The mold temperature rose rapidly in the first five seconds when heated [15]. The mold temperature then increased at a lower rate as its temperature was higher.

### 2.3. Characterization Technique

To investigate the characteristics of PP and ABS/TPU samples, besides the mechanical test, observing the fracture surface via scanning electron microscope (SEM) was also necessary to identify the fractured state [43,44]. The samples were tested via a fatigue test machine controlled using Labview NXG 5.0 and DOPSoft V4.00.11.18 software. Five samples were used to investigate each fatigue test condition. The PP samples were tested with a fixed amplitude of 0.2 mm, while the fatigue frequency varied from 1 to 5 Hz. The fatigue test stopped when the plasticity force decreases by 10 wt.% compared to the initial value. SEM microscope TM4000 (Hitachi, Japan) then observed the weld line surface. ABS/TPU samples conducted tensile tests via the tensile test machine AG-X Plus 20 kN (Shimadzu, Japan), at a 5 mm·min^−1^ speed.

## 3. Results and Discussion

### 3.1. Fatigue Test and Taguchi Analysis for PP

**Figure 4:**
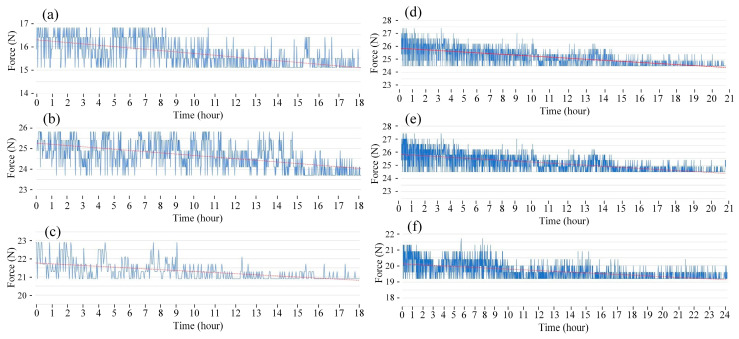
Fatigue diagrams of samples at 1 Hz at different heating times: (**a**) 0 s, (**b**) 3 s, (**c**) 6 s, (**d**) 9 s, (**e**) 12 s, and (**f**) 15 s.

**Figure 5:**
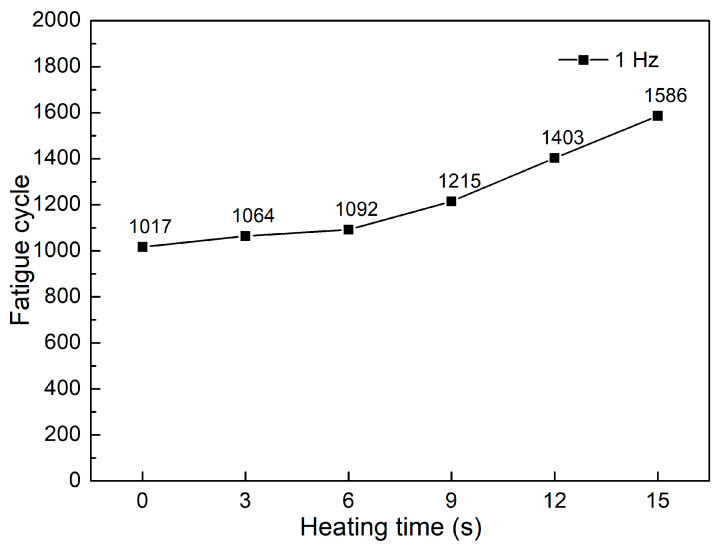
Average fatigue cycle of samples at 1 Hz at different heating times.

The fatigue diagrams and average fatigue cycle of samples at 1 Hz and various heating times are shown in Figure 4 and Figure 5. The sample with a pre-heated mold has a higher fatigue strength than the sample without mold heating. Furthermore, as heating time increases, the sample’s fatigue strength increases. When the heating time is increased from 0 to 15 s, the fatigue cycle improves from 1017 to 1586 times. Without heating, the fatigue cycle is 1017 times, which is consistent with Minh et al.’s [45] report. In that report, with the same test condition, the fatigue cycle result is 1058 times. Compared to the unheated mold, the fatigue cycle increases about 1.6 times at 15 s. The mold temperature values are 138.5, 160, 189.5, 201.3, and 219.1 °C, respectively, which correspond to 3, 6, 9, 12, and 15 s. Interestingly, at 15 s, the mold temperature is 219.1 °C, which is nearly the same as molten PP plastic at 220 °C. As a result, it is unnecessary to raise the mold temperature above the temperature of the molten plastic. In general, raising the mold temperature before injection increases the fatigue strength of the weld line. The weld line bonding becomes stronger during the filling stage because the molten plastic has a higher temperature.

**Figure 6:**
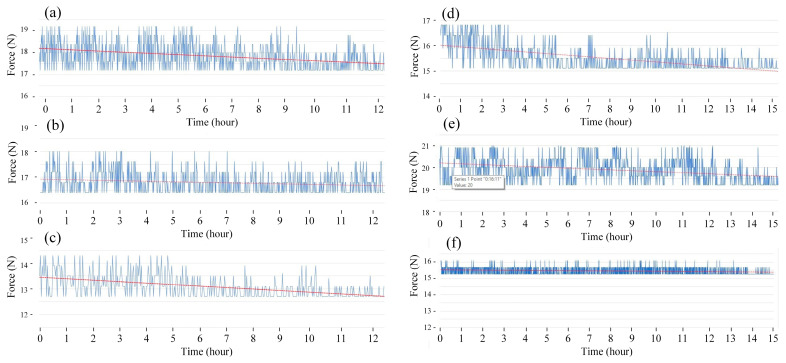
Fatigue diagrams of samples at 2 Hz at different heating times: (**a**) 0 s, (**b**) 3 s, (**c**) 6 s, (**d**) 9 s, (**e**) 12 s, and (**f**) 15 s.

**Figure 7:**
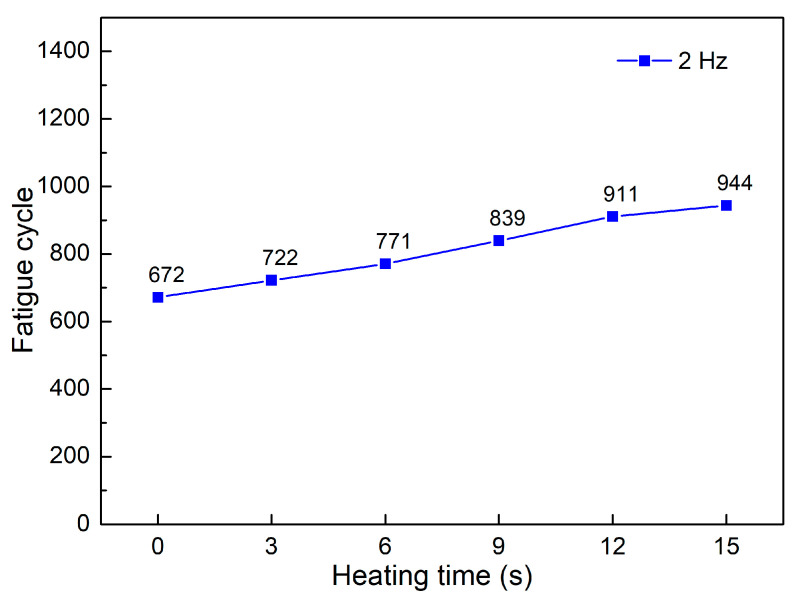
Fatigue cycle of samples at 2 Hz at different heating times.

Figure 6 and Figure 7 show the fatigue diagrams and the average fatigue cycle of samples at 2 Hz at different heating times. Increasing the heating time increases the fatigue cycle, which is a similar result in the 1 Hz case. The fatigue cycle gradually improves from 672 to 944 times when the heating time increases from 0 to 15 s, which is 1.4 times higher. The fatigue cycle in this 2 Hz frequency is lower than in 1 Hz, as shown in Figure 5 and Figure 7. In general, improving cycle times causes a decline in fatigue performance when the frequency is increased. The sample deforms more quickly when the vibration frequency is 2 Hz because the vibration time is two times longer. It is worth noting that despite the doubled deformation rate, the fatigue cycle does not shorten by a factor of two. This low rate of decreasing the fatigue cycle is due to the strengthening effect when the frequency is increased, which is consistent with the findings of Eftekhari et al. [36].

**Figure 8:**
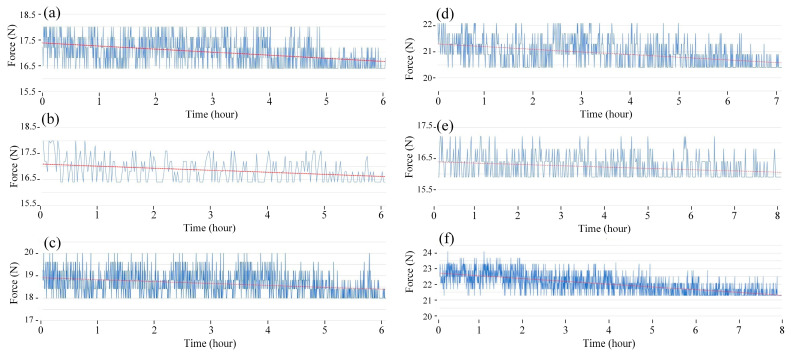
Fatigue diagrams of samples at 3 Hz at different heating times: (**a**) 0 s, (**b**) 3 s, (**c**) 6 s, (**d**) 9 s, (**e**) 12 s, and (**f**) 15 s.

**Figure 9:**
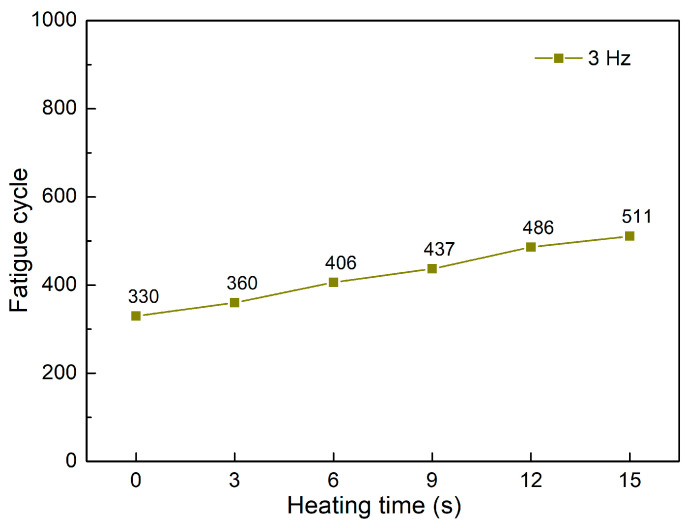
Fatigue cycle of samples at 3 Hz at different heating times.

Figure 8 and Figure 9 show the fatigue diagrams and the average fatigue cycle of samples at 3 Hz at different heating times. Similar to previous results, increasing the heating time leads to an increase in the fatigue cycle. The fatigue cycle gradually improves from 330 to 511 times when the heating time increases from 0 to 15 s, which is 1.5 times longer. The fatigue cycle is lower than in 1 and 2 Hz frequency cases. Therefore, the fatigue performance declines when the frequency is raised because of the improvement in the deformation times.

**Figure 10:**
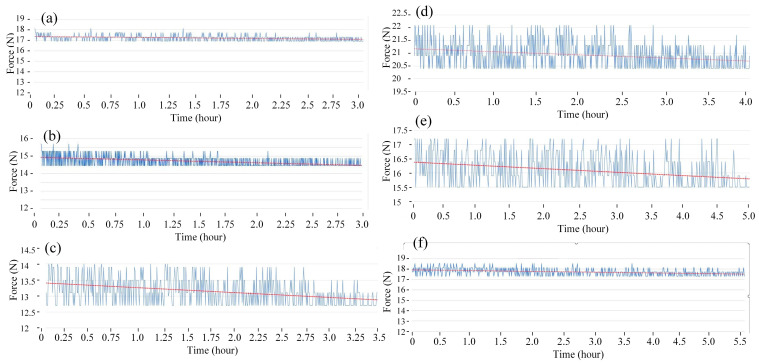
Fatigue diagrams of samples at 4 Hz at different heating times: (**a**) 0 s, (**b**) 3 s, (**c**) 6 s, (**d**) 9 s, (**e**) 12 s, and (**f**) 15 s.

**Figure 11:**
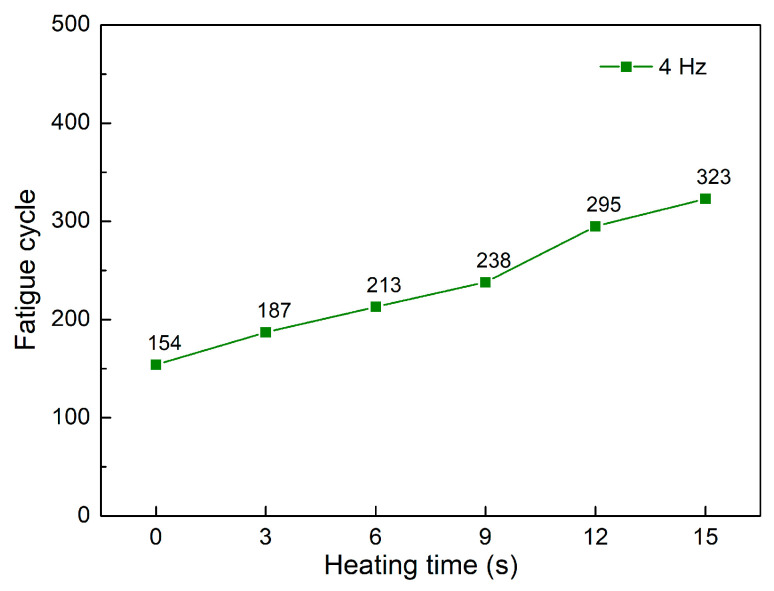
Fatigue cycle of samples at 4 Hz at different heating times.

Figure 10 and Figure 11 present the fatigue diagrams and the average fatigue cycle of samples at 4 Hz at different heating times. Similar to previous results, increasing the heating time leads to an increase in the fatigue cycle. The fatigue cycle gradually improves from 154 to 323 times when the heating time increases from 0 to 15 s, which is 2.1 times longer. This improving rate is higher than in 1–3 Hz cases. Raising the frequency causes a decrease in fatigue performance compared to instances with lower frequencies because the deformation rate is improving.

**Figure 12:**
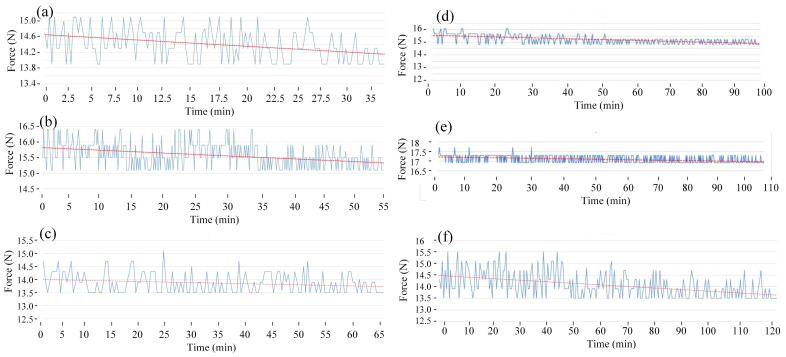
Fatigue diagrams of samples at 5 Hz at different heating times: (**a**) 0 s, (**b**) 3 s, (**c**) 6 s, (**d**) 9 s, (**e**) 12 s, and (**f**) 15 s.

**Figure 13:**
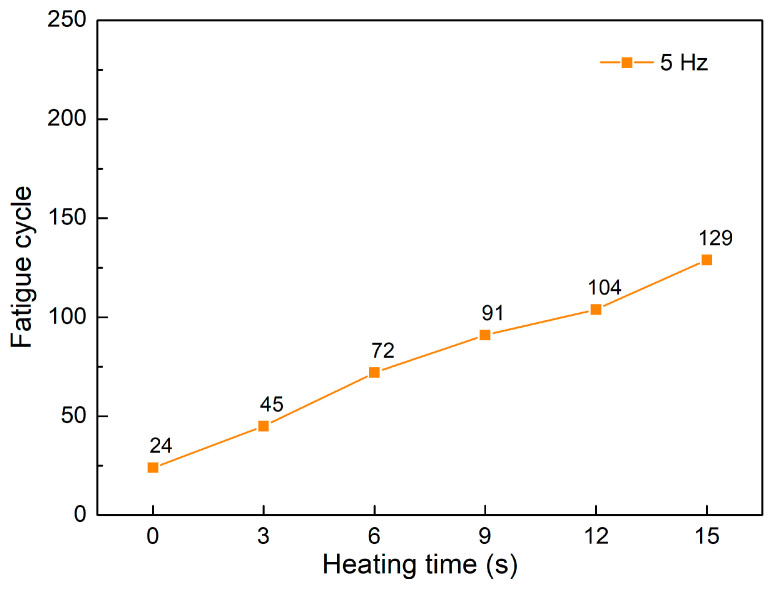
Fatigue cycle of samples at 5 Hz at different heating times.

**Figure 14:**
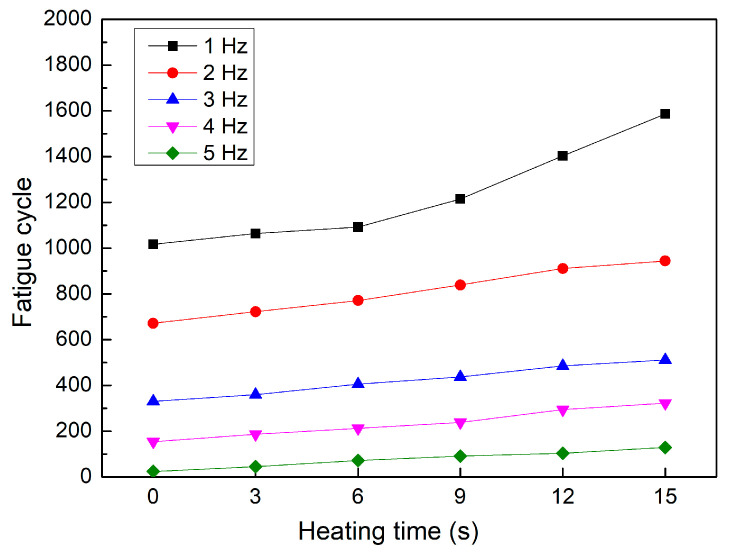
Fatigue cycle of samples at different frequencies and heating times.

Figure 12 and Figure 13 show the fatigue diagrams and the average fatigue cycle of samples at 5 Hz at different heating times. Similar to previous results, increasing the heating time leads to an increase in the fatigue cycle. The fatigue cycle gradually improves from 24 to 129 times when the heating time increases from 0 to 15 s, which is 5.4 times higher. Interestingly, the increasing rate is much higher in this case than in the 1–4 Hz cases, indicating the effectiveness of gas-assisted mold temperature control in improving fatigue strength. However, at 5 Hz frequency, the fatigue cycle is below 130 times, which is relatively low. The comparison between these five frequency levels is shown in Figure 14.

Figure 14 demonstrates the fatigue cycle diagram of PP samples tested at different heating times and frequencies. Increasing heating time generally leads to a steady improvement in fatigue strength. Interestingly, the curves at different frequencies are primarily parallel, indicating that the heating time consistently affects the fatigue strength of the samples. In the case of 1 Hz frequency, however, the increasing rate can be divided into two stages. The fatigue cycle increases more quickly from 6 to 15 s than in the previous stage. In addition, from 2 to 5 Hz, the fatigue strength improvement is more stable when raising the heating time. Compared to the sample without mold temperature control, the sample with mold heating at 15 s has 1.6, 1.4, 1.5, 2.1, and 5.4 times higher fatigue strength at frequencies of 1, 2, 3, 4, and 5 Hz. Furthermore, Zhou et al. [46] discovered that the PP sample without a weld line has a higher fatigue strength than the PP sample with a weld line.

**Figure 15:**
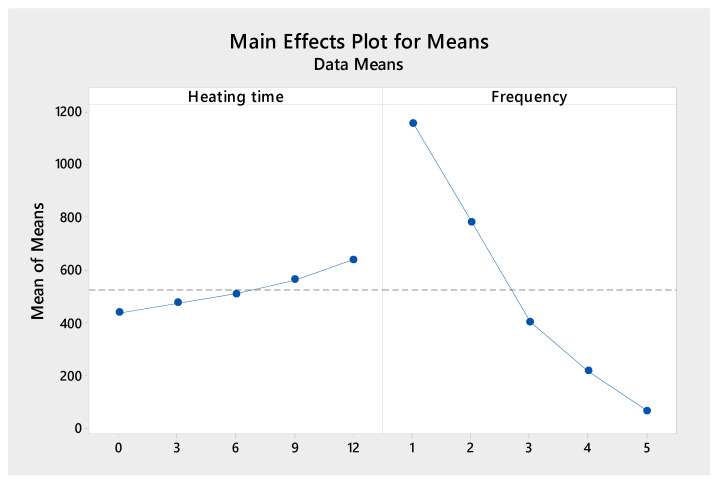
Main effects plot for means of fatigue cycle.

The Taguchi method is used to analyze the results. The Taguchi method is a mathematical and statistical strategy that combines control parameters and their relevant responses to improve the optimization process. The Taguchi method can be used to optimize the manufacturing process. It is also employed in experiment design, mainly when multiple factors are considered. The Taguchi method is commonly used in injection molding to investigate mechanical qualities with specified parameters [47,48,49]. Minitab 20.3 software, a L25 orthogonal array, two factors, and five levels were used in the analysis. Factor heating time had levels of 0, 3, 6, 9, and 12 s. Level 15 s was removed to include Taguchi design. Factor frequency has levels of 1, 2, 3, 4, and 5 Hz. Figure 15 shows the main effects plot for means of the fatigue cycle value with the “ larger is better” target and the response table for means. Minitab software exports this “the main effects plot for means” figure. The horizontal axis unit is seconds on the heating time subfigure, while on the frequency subfigure, it is hertz. Factor frequency rank is one, while the heating time is two, as shown in Table 2. Factor frequency impacts are more significant than the heating time factor. The influence of heating time and frequency on the fatigue strength is determined using multiple regression analyses. Using Taguchi’s experimental design, the regression equation is achieved using Minitab 20.3. The regression equation of the fatigue cycle value is:

Fatigue cycle = 1252.4 + 16.31 × Heating time − 274.8 × Frequency(1)

This equation presents the positive effect of the heating time and the negative effect of the frequency factor and TPU on the fatigue cycle values of the PP sample.

### 3.2. SEM Analysis for PP

**Figure 16:**
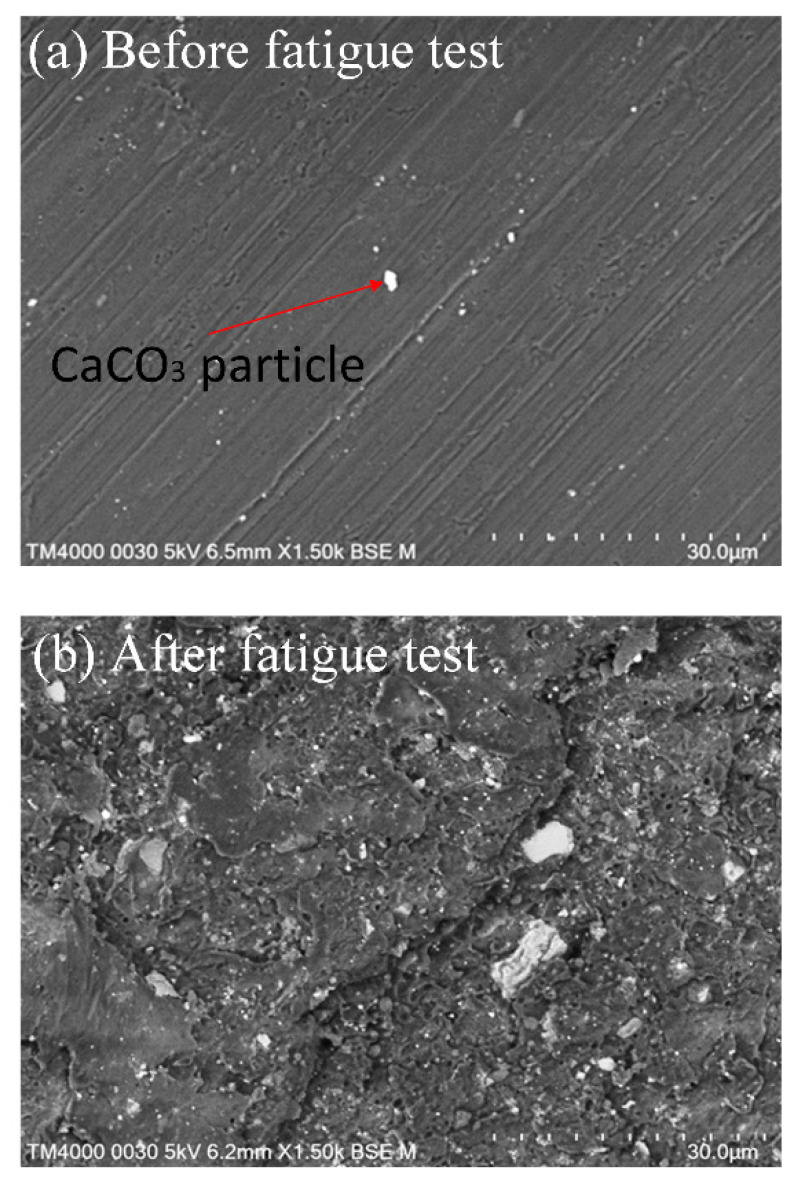
SEM microstructure of PP samples before and after fatigue test: (**a**) before testing, (**b**) after testing.

The SEM microstructure of PP samples before and after the fatigue test is shown in Figure 16. Before fatigue testing, the sample surface was smooth, as illustrated in Figure 16a. There are some CaCO_3_ particles on the sample surface, which are additives mixed with pure PP to enhance the mechanical properties. The sample is examined after testing on the weld line position, where the fatigue test has the most significant impact. The surface of the weld line is much rougher than the other areas, indicating weak bonding in this area. The surface becomes rougher, with microcracks pointing out the degradation of the plastics during the fatigue test, as presented in Figure 16b. Further impacts could cause these microcracks to accumulate and develop, leading to more severe failures [45,50].

### 3.3. Tensile Test and Taguchi Analysis for ABS/TPU Blends

This section examines the effects of TPU percentage and heating time. TPU percentages range at five levels, i.e., 10, 15, 20, 25, and 30 wt.%, while the heating time is selected at five levels, i.e., 0, 3, 6, 9, and 12 s. Each case has five samples for testing, and the average results are presented with standard deviations.

**Figure 17:**
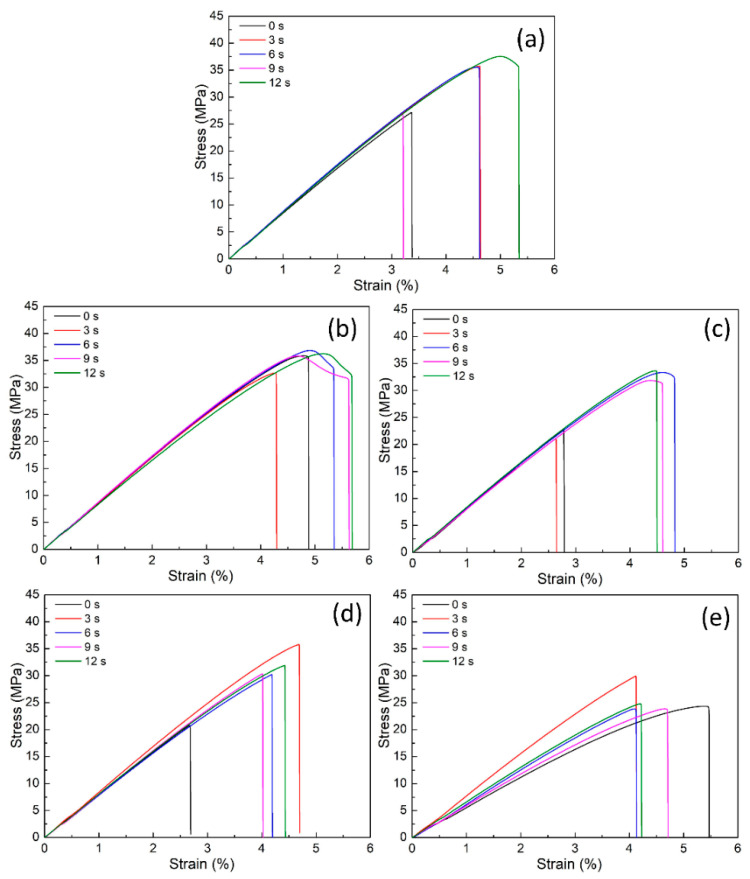
Stress–strain diagrams of ABS/TPU samples at different TPU percentages and heating times: (**a**) 10 wt.%, (**b**) 15 wt.%, (**c**) 20 wt.%, (**d**) 25 wt.%, and (**e**) 30 wt.%.

**Figure 18:**
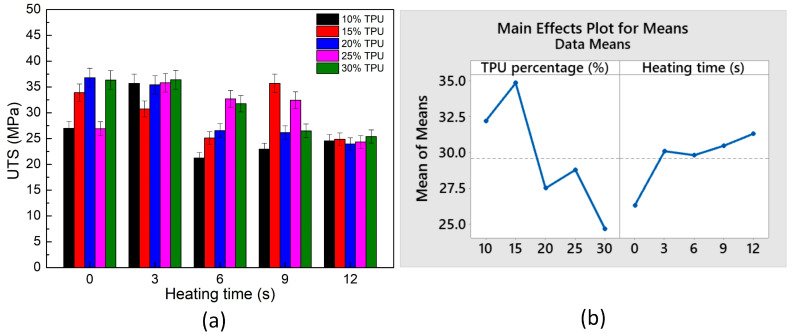
Average tensile strength of ABS/TPU samples at different TPU percentages and heating times and Taguchi analysis of UTS value: (**a**) UTS—heating time graph, and (**b**) main effects plot for means.

Figure 17 and Figure 18 present the stress–strain curves and the average ultimate tensile strength (UTS) values of the ABS/TPU blend samples at different TPU percentages and heating times. In Figure 18b, on the TPU percentage subfigure, the horizontal axis unit is wt.% TPU, while on the heating time subfigure, it is seconds. The UTS values range from 21.3 to 36.8 MPa. Chandra et al. [51] indicated that the UTS value of ABS is 32.76 MPa, which is close to the values found in this study. At the same time, Singh et al. [52] reported that the 3D-printed ABS/HIPS samples achieved a UTS value of 9.9–31.9 MPa. Singh et al. [53] said that the 3D-printed ABS/PLA/HIPS samples reached a UTS value of 6.97 MPa–10.78 MPa, which is relatively low—adding 15 wt.% TPU results in the highest UTS value, while 30 wt.% TPU lead to the blends reaching the lowest UTS value. This phenomenon could be explained based on the excellent mixture between the ABS and the TPU molecule when the TPU percentage is high enough. Due to their poor compatibility, the mixture becomes unstable when the TPU percentage is higher than 30 wt.%. Moreover, the average UTS value primarily increases when the heating time rises due to the better bonding formation in the weld line area. The effects of TPU percentage and the heating time are analyzed via the following Taguchi and ANOVA methods using Minitab software.

The Taguchi analysis is built based on TPU percentage and heating time, with five levels resulting in the L25 table. Despite using only two factors, Taguchi’s analysis helps visualize the meaning of the average table in Table 3. Figure 18b and Table 4 indicate that the TPU percentage has a higher impact rank with the UTS value than the heating time. The optimal UTS value is gained at 15 wt.% TPU and a heating time of 12 s, following the regression equation:

UTS = 35.97 − 0.422 *×* TPU + 0.345 *×* Heating time (2)

This equation also points out the positive coefficient of heating time; therefore, improving the heating time leads to a higher UTS value.

**Figure 19:**
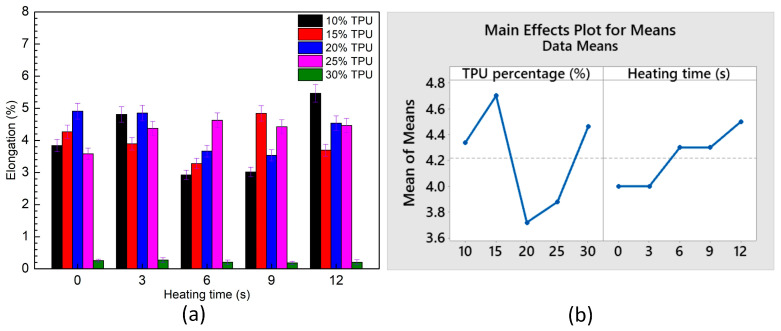
Average elongation of ABS/TPU samples at different TPU percentages and heating times and Taguchi analysis of UTS value: (**a**) Elongation—heating time graph, and (**b**) main effects plot for means.

Figure 19 and Table 5 and Table 6 show the average elongation of ABS/TPU samples for different TPU and PP percentages and Taguchi analysis. In Figure 19b, on the TPU percentage subfigure, the horizontal axis unit is wt.% TPU, while on the heating time subfigure, it is seconds. The elongation values range from 2.9 to 5.5 wt.%, as shown in Table 5. This result is similar to Dhakal et al.’s [54] report, with the 3D printed ABS sample values elongating in a range from 3.1 to 5.7 wt.%. Raising the heating time improves the elongation value due to a higher rate of weld line bonding. Taguchi analysis results prove that the TPU percentage has a higher impact rank with the elongation value than the heating time, as shown in Figure 19b and Table 6. The optimal elongation value is gained at 15 wt.% TPU and a heating time of 12 s, following the regression equation:

Elongation = 4.192 − 0.0116 *×* TPU + 0.0433 *×* Heating time (3)

This equation pinpoints the positive coefficient of heating time; therefore, improving the heating time leads to a higher UTS value.

**Figure 20:**
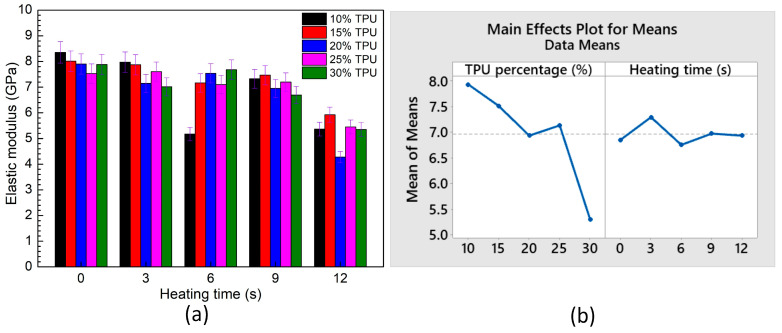
Average elastic modulus of ABS/TPU samples at different TPU percentages and heating times and Taguchi analysis of UTS value: (**a**) elastic modulus—heating time graph, and (**b**) main effects plot for means.

Figure 20 and Table 7 and Table 8 show the average elastic modulus of ABS/TPU samples for different TPU and PP percentages and Taguchi analysis. In Figure 20b, on the TPU percentage subfigure, the horizontal axis unit is wt.% TPU, while on the heating time subfigure, it is seconds. The elastic modulus values range from 4.3 to 8.4 GPa. Raising the TPU percentage leads to a decline in the elastic modulus value due to the ductility characteristic of TPU. Taguchi analysis results prove that the TPU percentage has a higher impact rank with the elastic modulus value than the heating time, as shown in Table 7 and Table 8. The optimal elastic modulus value is gained at 10 wt.% TPU and a heating time of 3 s, following the regression equation:

Elastic modulus = 9.264 − 0.1132 *×* TPU + 0.0053 *×* Heating time (4)

This equation pinpoints the positive coefficient of heating time; therefore, improving the heating time leads to a higher UTS value.

### 3.4. SEM Analysis for ABS/TPU Blends

**Figure 21:**
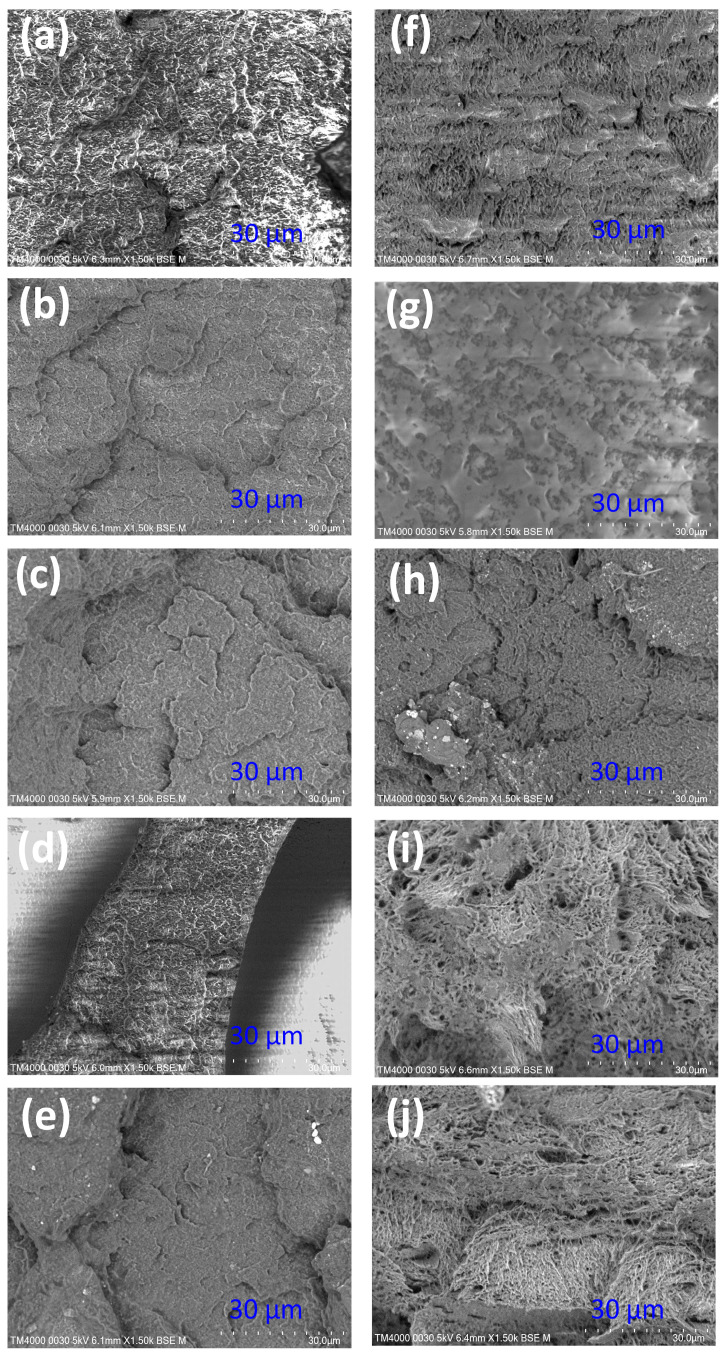
SEM picture of fracture surface of ABS/TPU samples: (**a**) 10 wt.% TPU—0 s, (**b**) 10 wt.% TPU—3 s, (**c**) 10 wt.% TPU—6 s, (**d**) 10 wt.% TPU—9 s, (**e**) 10 wt.% TPU—12 s, (**f**) 30 wt.% TPU—0 s, (**g**) 30 wt.% TPU—3 s, (**h**) 30 wt.% TPU—6 s, (**i**) 30 wt.% TPU—9 s, and (**j**) 30 wt.% TPU—12 s.

Two groups with 10 and 30 wt.% TPU are selected to observe the fracture surface’s microstructure. Figure 21 shows the SEM picture of the fracture surface. Figure 21a–e represents samples with 10 wt.% TPU in 0, 3, 6, 9, and 12 s. Similarly, Figure 21f–j corresponds to samples with 30 wt.% TPU in 0, 3, 6, 9, and 12 s. As ABS and TPU are different phases, the boundary between them is clear. The TPU area has a dimple shape on the fracture surface due to its high ductility, which is a result consistent with Azadi et al.’s [55] report. Moreover, the previous results show that samples with 30 wt.% TPU have lower UTS and elastic modulus values than samples with 10 wt.% TPU, while the elongation values are comparable. Therefore, the sample surface becomes rougher when increasing the TPU ratio.

## 4. Conclusions

This study examines the weld line strength of PP and ABS/TPU with the assistance of gas-assisted mold temperature control. The report surveys the effects of different heating times and frequencies on the fatigue strength of PP samples. In addition, the tensile strength of ABS/TPU samples at different TPU percentages and heating times are also examined. Some significant results that could be named are as follows:

— After heating, the mold temperature values are 138.5, 160, 189.5, 201.3, and 219.1 °C corresponding to 3, 6, 9, 12, and 15s. The fatigue strength of the sample increases upon increasing the heating time. Compared to the sample without mold temperature control, the sample with mold heating at 15 s has higher fatigue strength of 1.6, 1.4, 1.5, 2.1, and 5.4 times corresponding to 1, 2, 3, 4, and 5 Hz. Therefore, increasing the mold temperature before injection leads to a rise in the fatigue strength of the weld line. As the molten plastic has a higher temperature during the filling stage, the weld line bonding becomes stronger—the fatigue performance declines when improving the frequency due to the improvement in the cycle times. According to Taguchi’s analysis, factor frequency has a more significant influence on the fatigue strength of the PP sample than the heating time factor. The SEM results reveal that the surface of the weld line is much rougher than the other areas, indicating weak bonding in this area. The surface becomes rougher, with microcracks pointing out the degradation of the plastics during the fatigue test.
— The UTS values of the ABS/TPU blends range from 21.3 to 36.8 MPa. Adding 15 wt.% TPU results in the highest UTS value, while with 30 wt.% TPU, the blends reach the lowest UTS value. The elastic modulus values range from 4.3 GPa to 8.4 GPa. Taguchi’s analysis points out the positive coefficient of heating time; therefore, improving the heating time leads to higher UTS, elongation, and elastic modulus values. Moreover, the TPU percentage has a higher impact rank than the heating time on the mechanical properties of the ABS/TPU blends. The SEM results of the PP/TPU blends indicate that because ABS and TPU are different phases, the boundary between them is clear. The TPU area has a dimple shape on the fracture surface due to its high ductility. Moreover, the sample surface becomes rougher when increasing the TPU ratio.
— This study demonstrated the beneficial effect of gas-assisted mold temperature on the injection technique’s fatigue and tensile strength. The outcomes of this study could be applicable in various industries, ranging from automotive to consumer goods. In future work, the effects of vibration amplitude on the fatigue strength of the PP sample should be investigated. The effects of additives and samples without weld lines also need further consideration.

## Figures and Tables

**Table 1:** Molding conditions of testing samples.

Molding Parameters	Unit	Value for PP	Value for ABS/TPU Blend
Melt temperature	°C	220	220
Pre-heating time	s	0, 3, 6, 9, 12, 15	0, 3, 6, 9, 12
Mold temperature	°C	70, 138.5, 160, 189.5, 201.3, 219.1	70, 138.5, 160, 189.5, 201.3
Injection pressure	MPa	35	65
Injection time	s	2	2
Drying time (85 °C)	Hour	12	12
Holding time	s	0.5	0.8
Holding pressure	MPa	30	50
Injection speed	mm·s^−1^	40	35
Cooling time	s	20	20

**Table 2:** Response table for means.

Level	Heating Time	Frequency
1	439.40	1158.20
2	475.60	783.00
3	510.80	403.80
4	564.00	217.40
5	639.80	67.20
Delta	200.40	1091.00
Rank	2	1

**Table 3:** Average UTS values of ABS/TPU samples at different TPU percentages and heating times.

UTS (MPa)	0 s	3 s	6 s	9 s	12 s	Average
10% TPU	27.0 34 82	33.9	36.8	26.9	36.4	32.20
15% TPU	35.7 27.50	30.8	35.4	35.8	36.4	34.82
20% TPU	21.3	25.1	26.6	32.7	31.8	27.50
25% TPU	23.0	35.7	26.2	32.5	26.5	28.78
30% TPU	24.6	24.9	24.0	24.4	25.4	24.66
Average	26.322	30.08	29.80	30.46	31.30	

**Table 4:** Response table for means of UTS values of ABS/TPU samples at different TPU percentages and heating times.

Level	TPU	Heating Time
1	32.20 34 82	26.32
2	34.82 27.50	30.08
3	27.50	29.80
4	28.78	30.46
5	24.66	31.30
Delta	10.16	4.98
Rank	1	2

**Table 5:** Average elongation values of ABS/TPU samples at different TPU percentages and heating times.

Elongation (%)	0 s	3 s	6 s	9 s	12 s	Average
10% TPU	3.8	4.3	4.9	3.6	5.1	4.34
15% TPU	4.8	3.9	4.9	4.4	5.5	4.70
20% TPU	2.9	3.3	3.7	4.6	4.1	3.72
25% TPU	3.0	4.8	3.5	4.4	3.7	3.88
30% TPU	5.5	3.7	4.5	4.5	4.1	4.46
Average	4.0	4.0	4.3	4.3	4.5	

**Table 6:** Response table for means of elongation values of ABS/TPU samples at different TPU percentages and heating times.

Level	TPU	Heating Time
1	4.34	4.0
2	4.70	4.0
3	3.72	4.3
4	3.88	4.3
5	4.46	4.5
Delta	0.98	0.5
Rank	1	2

**Table 7:** Average elastic modulus values of ABS/TPU samples at different TPU percentages and heating times.

E (GPa)	0 s	3 s	6 s	9 s	12 s	Average
10% TPU	8.4	8.0	7.9	7.5	7.9	7.9
15% TPU	8.0	7.9	7.1	7.6	7.0	7.5
20% TPU	5.2	7.2	7.5	7.1	7.7	6.9
25% TPU	7.3	7.5	7.0	7.2	6.7	7.1
30% TPU	5.4	5.9	4.3	5.5	5.4	5.3
Average	6.8	7.3	6.8	7.0	6.9	

**Table 8:** Response table for means of elastic modulus values of ABS/TPU samples at different TPU percentages and heating times.

Level	TPU	Heating Time
1	7.94	6.86
2	7.52	7.30
3	6.94	6.76
4	7.14	6.98
5	5.30	6.94
Delta	2.64	0.54
Rank	1	2

## Data Availability

The data used to support the findings of this study are available from the corresponding author upon request.
